# Plant-Specific Myosin XI, a Molecular Perspective

**DOI:** 10.3389/fpls.2012.00211

**Published:** 2012-09-10

**Authors:** Motoki Tominaga, Akihiko Nakano

**Affiliations:** ^1^Molecular Membrane Biology Laboratory, RIKEN Advanced Science InstituteWako, Saitama, Japan; ^2^Japan Science and Technology Agency, PRESTOKawaguchi, Saitama, Japan; ^3^Department of Biological Sciences, Graduate School of Science, University of TokyoBunkyo-ku, Tokyo, Japan

**Keywords:** myosin XI, cytoplasmic streaming, intracellular transport, plants

## Abstract

In eukaryotic cells, organelle movement, positioning, and communications are critical for maintaining cellular functions and are highly regulated by intracellular trafficking. Directional movement of motor proteins along the cytoskeleton is one of the key regulators of such trafficking. Most plants have developed a unique actin–myosin system for intracellular trafficking. Although the composition of myosin motors in angiosperms is limited to plant-specific myosin classes VIII and XI, there are large families of myosins, especially in class XI, suggesting functional diversification among class XI members. However, the molecular properties and regulation of each myosin XI member remains unclear. To achieve a better understanding of the plant-specific actin–myosin system, the characterization of myosin XI members at the molecular level is essential. In the first half of this review, we summarize the molecular properties of tobacco 175-kDa myosin XI, and in the later half, we focus on myosin XI members in *Arabidopsis thaliana*. Through detailed comparison of the functional domains of these myosins with the functional domain of myosin V, we look for possible diversification in enzymatic and mechanical properties among myosin XI members concomitant with their regulation.

## Introduction

**Figure 1:**
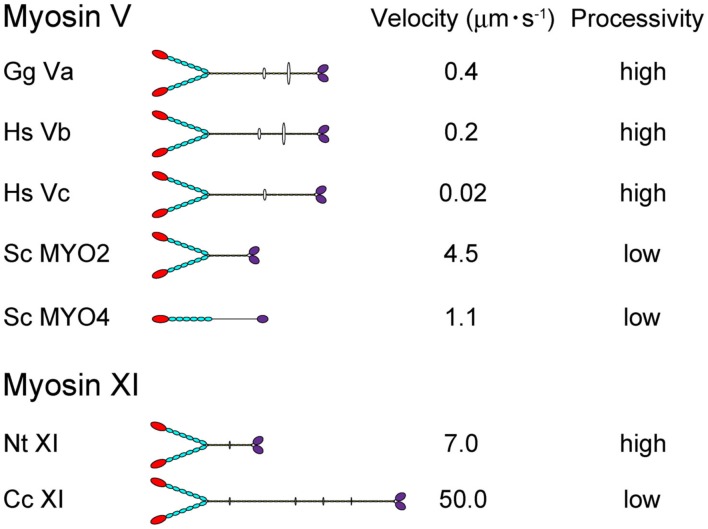
**The velocity and processivity of myosin Vs and myosin XIs characterized *in vitro***. Gg Va, *Gallus gallus* myosin Va (Mehta et al., 1999); Hs Vb, *Homo sapiens* myosin Vb (Watanabe et al., 2006); Hs Vc, *Homo sapiens* myosin Vc (Takagi et al., 2008; Watanabe et al., 2008); Sc Myo2 and 4, *Saccharomyces cerevisiae* myosin V (Reck-Peterson et al., 2001); Nt XI, *Nicotiana tabacum* 175-kDa myosin XI (Tominaga et al., 2003); and Cc XI, *Chara corallina* myosin XI (Ito et al., 2007).

Directional movement of motor proteins is essential for the regulation and maintenance of various biological phenomena through generation of motive force (Vale, 2003). Myosin is a molecular motor that moves along actin filaments using energy from ATP hydrolysis and is involved in various intracellular processes, including cell migration and adhesion; intracellular transport and localization of organelles, and macromolecules; signal transduction; and tumor suppression (Sellers, 2000). The myosin superfamily is divided into 37 classes (Richards and Cavalier-Smith, 2005; Foth et al., 2006). Among myosin species, the domain composition, molecular morphology, motile activity, and regulation of the protein are distinct (Krendel and Mooseker, 2005). In Figure 1, the velocity and processivity of myosin Vs and XIs that have been characterized *in vitro* are summarized. Although all are known as cargo transporters and have similar domain composition, their motile properties (e.g., velocity and processivity) are distinct. Such specific characteristics are considered to be closely related to the intracellular function of each myosin.

Plant myosins are classified into two plant-specific groups: classes VIII and XI. Myosin XI is morphologically similar to vertebrate myosin V and involved in organelle trafficking. In particular, the fast continuous intracellular transport, traditionally called cytoplasmic streaming is observed in plant species, ranging from algae to higher plants (Kamiya, 1981; Shimmen and Yokota, 2004; Shimmen, 2007). Angiosperms possess many genes of the class XI myosins. In contrast, class VIII myosin consists of fewer members. Molecular size of myosin VIII is smaller than that of myosin XI. The former has a shorter lever arm (consisting of three to four IQ motifs) and a shorter predicted coiled-coil domain. Several studies have indicated that myosin VIII is involved in new cell wall formation, intercellular transport through plasmodesmata and endocytosis (Reichelt et al., 1999; Baluska et al., 2001; Avisar et al., 2008a; Golomb et al., 2008; Sattarzadeh et al., 2008). These facts suggest that the functions of myosins XI and VIII are distinct at both the molecular and cellular levels.

In animal cells, microtubules are used for long-range transport, while actin filaments are used for shorter and local transport at the cell periphery. The analogy that has been made is that microtubules are the highways in the cell, while actin tracks are the secondary roads (Ross et al., 2008). On highways (microtubules), many types of vehicles (various classes of kinesins and a cytoplasmic dynein) equipped with diverse abilities (e.g., velocity, directionality, and cargo selectivity) work cooperatively (Caviston and Holzbaur, 2006). The orderliness of complex intercellular trafficking is presumably maintained through the regulation of, and interactions among, by these various motors.

In contrast, most plants have developed actin tracks as their highways. Surprisingly, the composition of vehicles running on actin tracks is very simple and consists of plant-specific myosins VIII and XI. This raises the question: Is plant intracellular trafficking simple? On the contrary, the number of proteins related to vesicle trafficking, such as Rabs and SNAREs, is even higher than that for other species, suggesting a very complex system (Saito and Ueda, 2009). In spite of the unique trafficking system comparable to that found in animals, progress in studies of plant myosin has been hampered because of difficulties in purifying myosin from plant tissue. Higher plant myosin XI with intact activity was first successfully isolated from lily pollen tubes and cultured tobacco BY-2 cells (Yokota and Shimmen, 1994; Yokota et al., 1999b). In combination with a single-molecule assay, it paved the way to reveal detailed properties of myosin XI at the molecular level.

Motile properties of a higher plant myosin XI, *Nicotiana tabacum* (Nt) 175-kDa myosin XI purified from cultured tobacco BY-2 cells, were first identified by optical trap nanometry at the single-molecule level. In brief, a single Nt 175-kDa myosin XI molecule moves processively toward the plus-end of an actin filament in 35 nm steps at 7 μm s^−1^. This is the fastest processive motor ever discovered (Tominaga et al., 2003). These characteristics suggest that Nt 175-kDa myosin XI is suitable for transporting cargo over a long distance with a small number of myosins at high velocity.

However, we should note that Nt 175-kDa myosin is just one myosin XI member. Fully sequenced angiosperm genomes have a dozen myosin XI genes, suggesting functional diversification among myosin XI members. The diversity of plant myosin XI members suggests that myosin is not merely the motive force for cytoplasmic streaming but is also involved in various biological processes accompanying force generation. In order to control the orderliness complex intracellular trafficking, spatial, and temporal regulation of myosin XI members equipped with diverse molecular functions would be expected. In this review, we focused on the enzymatic and mechanical properties of plant-specific myosin XI which is distinct from myosin V, and discussed the possible functional diversity among myosin XI members in Arabidopsis.

## Molecular Properties of Nt 175-kDa Myosin XI

### Molecular morphology

*Nicotiana tabacum* 175-kDa myosin XI expressed in cultured tobacco BY-2 cells has been extensively studied from the molecular to the cellular level (Yokota et al., 1999b, 2009, 2011; Tominaga et al., 2003, 2012). Sequence similarity of the motor domain of Nt 175-kDa myosin XI was 73% with *A. thaliana* (At) myosin XI-1 (MYA-1) and 84% with At myosin XI-2 (MYA-2) and 41% with myosin Va (*Mus musculus*).

**Figure 2:**
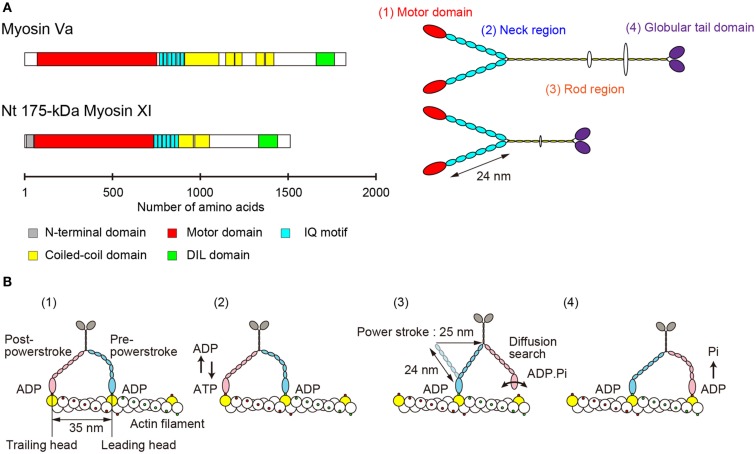
**(A)** Primary structure and morphology of myosin Va (*Mus musculus*) and Nt 175-kDa myosin XI (*Nicotiana tabacum*). **(B)** A general model for stepwise processive movement. (1) Both heads with ADP are strongly bound to actin subunits 35 nm apart (yellow). The trailing head (red) is in a post-powerstroke state, and the leading head (blue) is in a strained, pre-powerstroke state. (2) At physiological concentrations of ATP, ADP release from the trailing head is the rate-limiting step of the mechanochemical cycle. After ADP release, a new ATP molecule binds to the trailing head. (3) Immediately, the trailing head detaches from the actin filament. The strain stored in the neck domain of the leading head swings the detached head forward, generating a 25-nm power stroke. The new leading head (bound to ADP-Pi) finds a binding site 35 nm forward via a diffusion search. (4) After binding to actin, the leading head rapidly releases Pi and establishes a strong binding conformation, the same state as in (1). The strain and the biased diffusion search of the detached head and its subsequent rebinding constrain the movement of a myosin molecule to a single direction, i.e., toward the plus-end of actin.

The primary structure of Nt 175-kDa myosin XI predicted a morphological similarity to that of myosin Va (Espreafico et al., 1992; Walker et al., 2000). Nt 175-kDa myosin XI can be divided into four major structural domains (Tominaga et al., 2003). (1) The N-terminal motor domain containing the actin-binding site and nucleotide-binding site. (2) The neck region, an extended α-helical lever arm consisting of six IQ motifs, which is stabilized by binding to the light-chain calmodulin (CaM) or CaM-related proteins. (3) The rod region containing an α-helical coiled-coil responsible for dimerization of the molecule. In the center of this domain, 10 amino acids break the α-helical coiled-coil structure. (4) The C-terminal globular tail domain (GTD), which binds adapter proteins that link myosin to the cargo (Figure 2A).

Electron microscopy of rotary-shadowed Nt 175-kDa myosin XI confirmed these predictions. There are two head domains, each attached to a long neck of approximately 24 nm in length. The two neck domains combine at a thin stalk that extends for approximately 25 nm. Two GTDs are found at the C-terminus of the molecule’s putative cargo binding domain (Tominaga et al., 2003). Although the domain composition and morphological features are similar to those of vertebrate myosin V (Figure 2A), the motile properties are distinct, as described in the following sections.

### Velocity and directionality

The velocity of myosin XI directly reflects the velocity of cytoplasmic streaming, because this process is generated by sliding of myosin XI associated with organelles along actin cables. The velocity of Nt 175-kDa myosin XI was characterized by a conventional *in vitro* motility assay in which fluorescently labeled actin filaments glide over glass surfaces coated with myosin in the presence of ATP (Kron et al., 1991). The V_max_ of the actin sliding velocity on Nt 175-kDa myosin XI was approximately 5 μm s^−1^, which is comparable with V_max_ of the velocity of cytoplasmic streaming observed in higher plant cells (Yokota et al., 1999b; Tominaga et al., 2003) and 10-fold higher than V_max_ of the actin sliding velocity on myosin Va under similar conditions (Mehta et al., 1999). The velocity of At myosin XI-1, which is a motive force for rapid organelle movement in Arabidopsis, was also confirmed by an *in vitro* motility assay. The recombinant motor domain of At myosin XI-1 connected to an artificial lever arm composed of α-actinin translocated actin filaments at a maximum velocity of 1.8 μm s^−1^. This value corresponded to a motility of 3.2 μm s^−1^ for full-length At myosin XI-1, taking into account the difference in the lever arm length (Hachikubo et al., 2007). The velocity of *Chara corallina* myosin XI can reach to approximately 50 μm s^−1^, which is comparable to the velocity of cytoplasmic streaming in Chara internodal cells. This myosin is known as the fastest motor protein in the living world (Figure 1). Detailed biochemical analysis revealed that this high velocity is attributable to its motor domain which has high ATPase activity and rapid ADP dissociation from actin-myosin (Ito et al., 2003, 2007, 2009). Because Chara internodal cells attain large size (>10 cm), this fast cytoplasmic streaming would be necessary for distribution of materials within the cell (Verchot-Lubicz and Goldstein, 2010). In Arabidopsis, simultaneous knock-out of several myosin XI members (XI-1, XI-2, XI-K, and XI-I) demonstrated a defect in the development of the plant concomitant with the inhibition of organelle movement (Peremyslov et al., 2010; Ojangu et al., 2012). These results suggested that intracellular movement driven by myosin XI is closely related to the development of the plant. However, the relationship between cytoplasmic streaming and plant cell size has not been demonstrated irrefutably.

The directionality of myosin can be determined by an *in vitro* motility assay using plus-end marked actin filaments (Wells et al., 1999). Nt 175-kDa myosin XI translocated actin filaments with the minus-end leading, showing that Nt 175-kDa myosin XI is a plus-end directed myosin (Tominaga et al., 2003).

### Processivity

Myosin Va was the first myosin identified as a processive motor. Processivity means that it takes multiple steps on its track before dissociating (Mehta et al., 1999).

Several parameters for assessing the processivity of Nt 175-kDa myosin XI were extracted from kinetic and motility assays (Tominaga et al., 2003). (1) No reduction in actin velocity was observed as the myosin XI surface density was decreased to 50 molecules μm^−2^. (2) A plot of the attachment rate as a function of myosin surface density was a good fit to the theoretical relationship for a single myosin molecule being sufficient for attachment and motility of actin filaments. (3) Actin filaments observed at very low myosin surface densities rotated erratically about a vertical axis through a fixed point on the surface, where a single myosin molecule is presumably located. (4) Kinetic processivity, defined as the average number of ATPase cycles before dissociation of motors (myosin) from their track (actin): a value of >10^7^ M^−1^ s^−1^ indicates many ATPase cycles per diffusional encounter between myosin and F-actin; for myosin XI, it was 7.9 × 10^6^ M^−1^ s^−1^, suggesting a processive motor. (5) The duty ratio for Nt 175-kDa myosin XI, which is the average proportion of time that a head spends bound during an average catalytic cycle period, was 0.81, again suggesting that this myosin XI is a processive motor. Coupled with the single-molecule analysis described in Section “Step Size” below, Nt 175-kDa myosin XI was defined as the fastest known processive motor. At myosin XI-1 also has a high duty ratio (0.7), suggesting that At myosin XI-1 is a processive motor (Hachikubo et al., 2007). On the other hand, the duty ratio of Chara myosin XI is very low (0.3), suggesting that it is non-processive, although it is also a motive force for cytoplasmic streaming (Awata et al., 2003; Kimura et al., 2003). Differences in processivity are also found among class V myosins. Vertebrate myosin Va, b, and c are processive (Mehta et al., 1999; Watanabe et al., 2006, 2008; Takagi et al., 2008), whereas the processivity of MYO2 (myosin V of *Saccharomyces cerevisiae*) is low (Reck-Peterson et al., 2001; Figure 1). Although the significance of processivity for intracellular trafficking remains unclear, the following hypothesis can be proposed: If the processivity is high, it is expected that the motor can effectively transport a small organelle or vesicle with a small number of motor molecules. On the other hand, if the processivity is low, the motor can effectively transport a large organelle with many motor molecules. Thus, as a result of the shorter duration of the state of strong binding to actin, the physical interference by neighboring motors is decreased or sufficient velocity is generated with minimal energy consumption. Processivity may be optimized for the specific cargo carried by each motor.

### Step size

In order to elucidate the mechanism underlying the processive movement of Nt 175-kDa myosin XI on actin, the movement of single myosin XI molecules was measured in a bead assay using an optical trap (Kojima et al., 1997; Sakakibara et al., 1999). Detailed examination of bead movement revealed that single myosin XI molecules moved along an actin filament with 35 nm steps, corresponding closely to the 35 nm pseudorepeat of the actin helix and the step size of myosin Va. The maximum velocity was determined to be 7 μm s^−1^, which is 10-fold higher than that of myosin Va, and this exemplifies the fastest known processive movement. The mean maximal force was approximately 0.5 pN (Tominaga et al., 2003), which is considerably less than the mean maximal force observed for myosin Va (3.0 pN).

The lever arm swing model is widely accepted as an explanation for the stepwise processive movement of myosin Va (Hammer I and Sellers, 2012; Figure 2B). Several features are necessary for achieving such processive movement. (1) One head must remain bound to the actin filament at all times. The motor domain must therefore have a high duty cycle, meaning that it spends most of its time attached to actin in a strongly bound state (De La Cruz et al., 1999). (2) Dimer formation must occur to generate hand-over-hand motion (Purcell et al., 2002). (3) The neck domain must have sufficient length in order to attach to the next binding site on the pseudorepeat of the actin helix because sites on the same azimuth are spaced at 35 nm intervals (Sakamoto et al., 2003; Oke et al., 2010). (4) Strain-dependent changes in the kinetics of the two heads are accomplished through the long lever arms that join the two motor domains. Intramolecular strain can accelerate the rate of ADP release from the trailing head, inhibiting the rate of ADP release from the leading head. This prevents dissociation of the leading head before the dissociation of the trailing head (Veigel et al., 2002).

Through biochemical, biophysical, and electron microscopic studies, we concluded that Nt 175-kDa myosin XI is highly specialized in order to produce the fastest known processive movement while concomitantly generating low forces. The high velocity can be explained by high ATPase turnover in the motor domain, which was 95 s^−1^ for Nt 175-kDa myosin XI and 12 s^−1^ for myosin Va. The rate-limiting step under physiological conditions may be ADP release. According to hydrodynamic analysis (Yoneda and Nagai, 1988), the motive force of cytoplasmic streaming per unit interface was 0.3 ± 0.4 pN μm^−2^, which is comparable to our results, given the assumption that a spherical organelle of approximately 1 μm in diameter is driven by a few myosin molecules. Performance characteristics of myosin Va and Nt 175-kDa myosin XI are summarized in Table 1.

**Table 1:** **Comparison of molecular properties of myosin Va and Nt 175-kDa myosin XI**.

	Velocity (μm s^−1^)	ATPase activity (pi·head^−1^ s^−1^)	Force (pN)	Step size (nm)	Processivity
Myosin Va (*Gallus gallus*)	0.3	3–15	3	36	High
Myosin XI (*Nicotiana tabacum*)	5.0–7.0	95	0.5	35	High

## Plant Myosin XI Members in Arabidopsis

Angiosperms generally possess approximately 20 myosin genes that belong to the plant-specific classes VIII and XI. For example, *Brachypodium distachyon*, *A. thaliana*, and *Oryza sativa* respectively have 2, 4, and 2 class VIII and 9, 13 and 12 class XI genes (Reddy, 2001; Jiang and Ramachandran, 2004; Peremyslov et al., 2011), with remarkable diversification in class XI. Such diversity among myosin XI members suggests that these proteins are not merely a redundant motive force for cytoplasmic streaming but are also involved in various organelle-specific movements or other biological processes. Development of green fluorescent protein (GFP) and its variants enabled visualization of individual organelles, such as the endoplasmic reticulum, mitochondria, peroxisomes, and Golgi stacks in living plant cells. Live cell imaging of each organelle revealed that the movement was not only continuous along the direction of flow of cytoplasmic streaming, but also more complex and specific depending on the organelles, which moved with various velocities and in various directions with temporary pauses (Hamada et al., 2012). Cell biological approaches using knock-out mutants, RNAi, or overexpression of the myosin tail (dominant-negative form) demonstrated the involvement of particular myosin XI members (XI-1, XI-2, XI-B, XI-C, XI-E, XI-I, and XI-K) in organelle transport in Arabidopsis (Avisar et al., 2008b, 2009, 2012; Peremyslov et al., 2008, 2010; Prokhnevsky et al., 2008; Sparkes et al., 2008; Ueda et al., 2010). Generation of such distinct organelle movements could only be achieved by myosin XI members equipped with distinct molecular properties. In order to understand the plant-specific actin–myosin system, it will be essential to characterize each myosin XI member at the molecular level.

**Figure 3:**
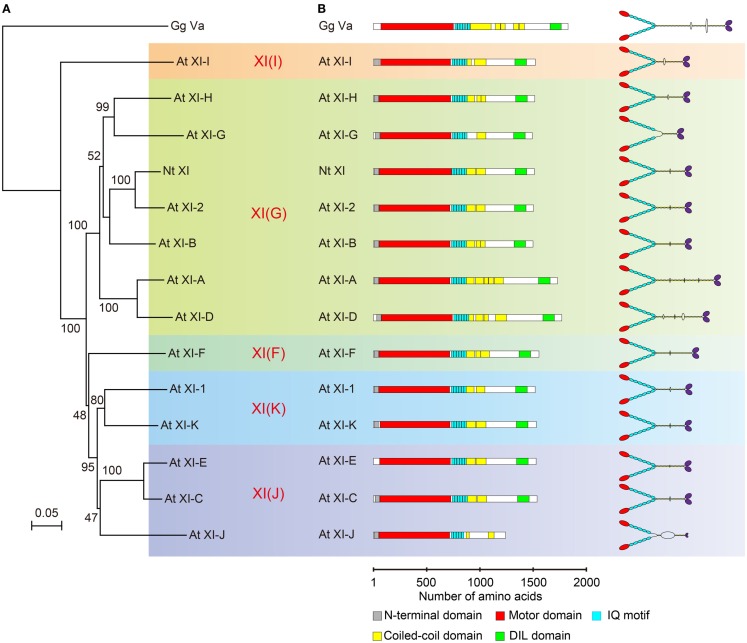
**(A)** Phylogenetic tree for the motor domain of plant myosin XIs. Numbers indicate percentage bootstrap value of 10,000 trials. Each color represents lineages determined by Avisar et al. (2008b). **(B)** Primary structure and putative morphology of At myosin XI members. Gg Va, *Gallus gallus* myosin Va; Nt XI, *Nicotiana tabacum* 175-kDa myosin XI; and At XI, *Arabidopsis thaliana* myosin XI.

Phylogenetic analysis of plant myosin indicated that myosin XI split into five lineages (Avisar et al., 2008b; Peremyslov et al., 2011). According to this classification, the phylogenetic tree for the motor domain of At myosin XI members and Nt 175-kDa myosin XI was partitioned by colors for each lineages (Figure 3A). This revealed that Nt 175-kDa myosin XI is closely related to At myosin XI-2, both of which are responsible for the translocation of the endoplasmic reticulum (Ueda et al., 2010; Yokota et al., 2011). At myosin XI-1 belongs to a clade different from that of Nt 175-kDa myosin XI, although they have similar motile properties *in vitro*. At present, because the relationship between amino acid composition and motile properties of myosin (such as velocity and processivity) remains unclear, coupled with limited information from biochemical approaches, the prediction of molecular properties of each myosin XI member based on phylogenetic analysis is difficult.

Although biochemical and mechanical analyses are essential to confirm molecular properties, it is possible to predict the diversity of myosin XI members through comparison of each functional domain with well-characterized myosins (e.g., myosin V). In the second half of this review, we focus on myosin XI members in Arabidopsis, and try to find clues for the putative diversity of enzymatic and mechanical properties concomitant with their regulation.

### Molecular morphology

The predicted domain composition and molecular morphology of all myosin XI members in Arabidopsis are illustrated in Figure 3B. Macroscopically, except for the length of the rod region, the domain composition, and morphological features of myosin XI members are similar. As described in the first half of this review, the molecular properties of myosin cannot be judged by morphological features.

### Motor domain (actin-binding interface)

In the motor domain, most myosins have positively charged surface loops, called loops 2 and 3, at the actin-binding interface. These loops are involved in the initial weak electrostatic interaction with negatively charged residues in subdomain 1 of actin (Mornet et al., 1979; Yamamoto and Sekine, 1979). Many studies have revealed that positively charged loops are involved in actin-activated ATPase activity, affinity for actin, velocity, and processive run length (Uyeda et al., 1994; Furch et al., 1998; Goodson et al., 1999; Knetsch et al., 1999; Joel et al., 2003; Yengo and Sweeney, 2004; Hodges et al., 2007; Ito et al., 2009). Because the length and amino acid composition of these loops are highly variable among classes of myosins, it has been suggested that the diverse enzymatic and motile activities of myosins are achieved in part through these variations (Spudich, 1994; Goodson et al., 1999).

Loop 2 of plant-specific myosin XI is shorter than that of other myosin classes. Loop 2 consists of 18 amino acid residues in Nt 175-kDa myosin XI, 28 residues in skeletal muscle myosin II, and 45 residues in myosin Va. At myosin XI members also have a short loop 2 with 17–19 residues. The functional significance of the short loop 2 in plant myosin XI remains unclear.

The composition of charged residues in loops 2 and 3 varies among myosin XI members, showing a net charge ranging from 0 to +2 in loop 2 and +1 to +3 in loop 3 (Table 2). This net charge variation suggests a difference in enzymatic and motile activities among myosin XI members. The relationship between the loop charge and myosin motility will be examined by investigating the motile properties of each myosin XI member *in vitro*.

**Table 2:** **Sequences of loops 2 and 3 of myosin**.

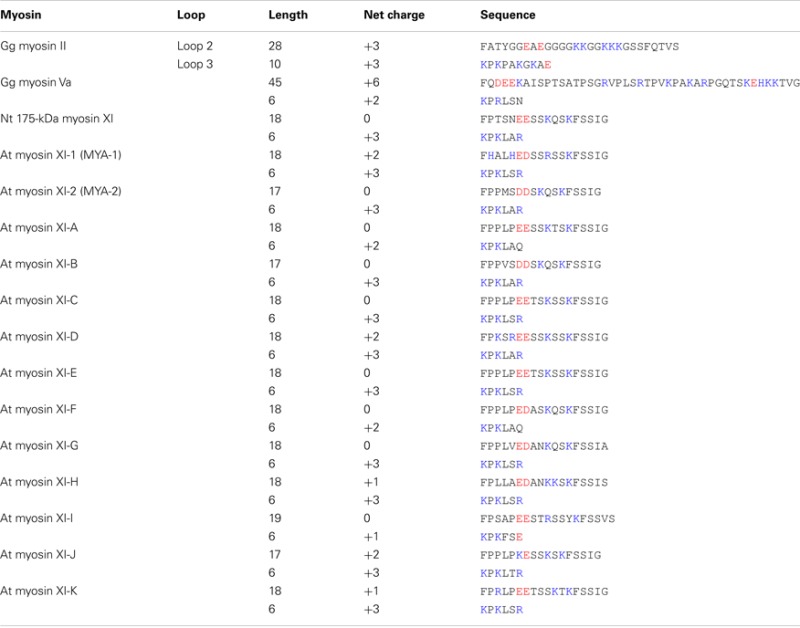

*Acidic and basic residues are highlighted in red and blue, respectively. Gg, *Gallus gallus*; Nt, *Nicotiana tabacum*; and At, *Arabidopsis thaliana**.

### Neck region

Similar to myosin Va, myosin XI members have a long neck region consisting of six IQ motifs with the consensus sequence IQXXXRGXXXXR, where X denotes any amino acid (Bahler and Rhoads, 2002).

In myosin Va, each motif binds to the light chains of CaM or CaM-related proteins that have not retained functional Ca^2+^-binding sites. Binding of the IQ motif to the light chain confers rigidity to the neck and allows it to act as a lever arm. A small change in the catalytic motor domain induced by ATP hydrolysis is amplified to a large displacement through a relatively rigid neck domain (Uyeda et al., 1996). Because this mechanical change corresponds to the power stroke, the velocity of myosin is proportional to the length of the neck (Sakamoto et al., 2003, 2005). In addition, a sufficiently long neck domain is essential for the processive run of myosin V. This allows myosin V to move hand-over-hand with a long (35 nm) stride on the binding sites of the pseudorepeat of the actin helix (Figure 2B).

For these reasons, the number of the light chain and their composition is important in determining the velocity and processivity of myosin XI. The amino acid sequence in each IQ motif of myosin XI members is distinct, suggesting diversity in the lever arm function, including Ca^2+^–CaM regulation as described in Section “Regulation by Ca^2+^ and CaM.”

### Rod region

The rod region consists of a predicted coiled-coil sequence that lies between the IQ motifs and the globular tail. It forms an α-helical coiled-coil allowing the myosin molecule to dimerize. However, the existence of a predicted coiled-coil sequence does not guarantee stable dimerization of myosin. Several biochemical and structural studies have revealed that MYO4 (myosin V of *S. cerevisiae*; Figure 1), myosins VI, VII, and X exist as monomers, although they have a predicted coiled-coil sequence (Wu et al., 2002; Lister et al., 2004; Knight et al., 2005; Dunn et al., 2007; Hodges et al., 2008; Spink et al., 2008; Umeki et al., 2009). In these myosins, monomer–dimer transition is proposed as a regulatory mechanism for intracellular transport. Two MYO4p–She3p complexes are recruited by the tetrameric She2p to form a double-headed complex (Krementsova et al., 2011). Cargo proteins, such as Dab2 and optineurin, directly induce myosin VI dimerization (Phichith et al., 2009). A MyRip–Rab27a complex facilitates myosin VIIA monomers binding on membrane vesicles, then dimerizes them when they are closely clustered on the vesicle. (Sakai et al., 2011). Binding of phosphatidylinositol-3,4,5-triphosphate to the PH domain induces the formation of the myosin X dimer (Umeki et al., 2011).

The predicted coiled-coil sequence and length of the rod region of myosin XI are highly variable among members, as illustrated in Figure 3B. For example, the predicted coiled-coil region in At myosins XI-G and XI-J is narrow relative to that in other members, suggesting that these myosin XIs exist in a monomeric form.

### Regulation by Ca^2+^ and CaM

**Figure 4:**
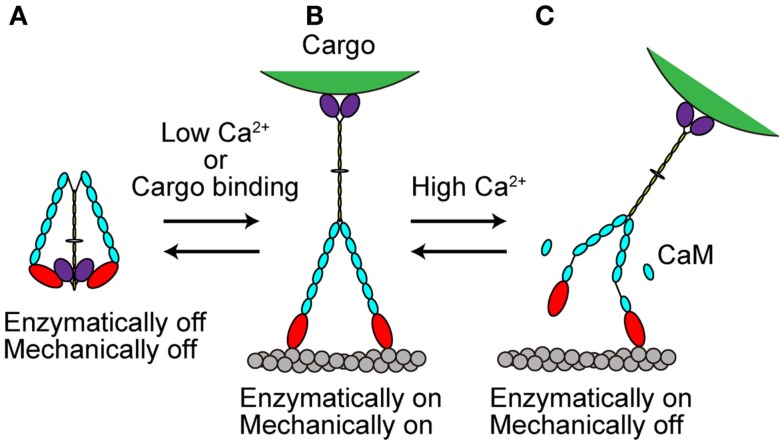
**Regulation of myosin Va**. **(A)** When cargo is not present, myosin Va adopts a folded, inhibited conformation in the absence of Ca^2+^. **(B)** At low Ca^2+^ concentrations or with cargo binding in the absence of Ca^2+^, myosin Va becomes unfolded and active. **(C)** At high Ca^2+^ concentrations, the processive movement of myosin Va is inhibited via dissociation of CaM molecules.

The *in vivo* functions of various types of myosins are controlled both temporally and spatially. This control is partly accomplished by the regulation of myosin activity at the molecular level. Several recent studies have revealed that myosin Va activity is regulated in at least two ways, as described in the following sections (Figure 4). The processive movement of myosin Va is inhibited at high Ca^2+^ concentrations via dissociation of one or two CaM molecules (Krementsov et al., 2004; Li et al., 2004; Nguyen and Higuchi, 2005; Lu et al., 2006). A truncation assay revealed that Ca^2+^-induced CaM dissociation occurs at the second IQ domain (Koide et al., 2006; Trybus et al., 2007; Figure 4C).

*In vitro* motility and immunological studies revealed that the motility of higher plant myosin XI is also negatively regulated through Ca^2+^-induced CaM dissociation at Ca^2+^ concentrations of >10 μM (Yokota et al., 1999a,b). The mechanism of inhibition of myosin XI by Ca^2+^ has been investigated at the single-molecule level using Nt 175-kDa myosin XI. At high Ca^2+^ concentration, the affinity between actin filaments and Nt 175-kDa myosin XI decreased concomitantly with the detachment of CaM light chains from the neck domains. Electron microscopic observations showed that the neck domain of Nt 175-kDa myosin XI shortened by 30% in the presence of Ca^2+^. Single-molecule analysis revealed that the step size of Nt 175-kDa myosin XI in the presence of Ca^2+^ was shortened to 27 nm under low load and to 22 nm under high load, compared with 35 nm independent of load in the absence of Ca^2+^ (Tominaga et al., 2012). Although Ca^2+^-induced CaM dissociation is a common mechanism for controlling the processive movement of vertebrate myosin Va, the effect on processivity is different, perhaps because of the site of CaM dissociation. These results suggest that Ca^2+^ regulation may be variable and is optimized depending on the myosin species and the specific organelle to be transported *in vivo*. The amino acid composition of the IQ motif of myosin XI members is highly variable, while the number of CaMs and the site of CaM dissociation on the IQ motif remain unclear. A study of the crystal structure of myosin Va revealed that the non-consensus residues are essential for the exact positioning of CaM, resulting in different interactions with CaM along the lever arm (Houdusse et al., 2006). Considering the mechanical function of the neck domain, it is plausible that even under the same Ca^2+^ conditions, CaM dissociation affects the motile activity of myosin XI members differently, depending on the number and location of CaMs involved.

It is uncertain whether such a high Ca^2+^ concentration (>10 μM) can be physiologically achieved in plant cells. A recent study showed the existence of Ca^2+^ microdomains with transiently elevated Ca^2+^ concentrations (ranging >100 μM) in a small region (Rizzuto and Pozzan, 2006) within animal cells. Such a local elevation of Ca^2+^ concentrations has also been reported within plant cells (Kudla et al., 2010). Thus, it is possible that myosin XI movement is regulated locally by Ca^2+^, without a global elevation of cytoplasmic Ca^2+^ levels. The level of regulation might also differ among myosin XI members depending on the Ca^2+^ sensitivity of each neck region.

### The inhibited state

Recently, several studies have revealed that full-length myosin Va adopts a folded, inhibited conformation in the absence of both cargo and Ca^2+^ (Figure 4A). At low Ca^2+^ concentrations or when the cargo binds in the absence of Ca^2+^, myosin Va becomes unfolded and active (Krementsov et al., 2004; Li et al., 2004, 2006; Wang et al., 2004; Ikebe et al., 2005; Liu et al., 2006; Lu et al., 2006; Thirumurugan et al., 2006; Figure 4B).

Myosin Va is both enzymatically and mechanically “off” when folded into a compact structure, and “on” when unfolded to an extended structure. Inhibition is due to the electrostatic binding of GTDs to motor domains, which may prevent the motor from completing its enzymatic cycle by interfering with the release of hydrolysis products from the catalytic site (Olivares et al., 2006; Sato et al., 2007). A conserved acidic residue in the motor domain (Asp-136) and two conserved basic residues in GTD (Lys-1706 and Lys-1779) were identified as critical residues for this regulation (Li et al., 2008). Two basic residues (Arg-1359 and Arg-1434) in the GTD of At myosin XI-1 have been predicted to be involved in such an electrostatic interaction (Li and Nebenfuhr, 2007). These same residues (Arg-1368 and Arg-1443) in the GTD of At myosin XI-K were found to be necessary for the dominant-negative effect of tail overexpression, indicating the existence of head–tail interaction (Avisar et al., 2012). These two basic residues in GTD are well conserved among At myosin XI members, while the acidic residue in the motor domain of myosin Va is not found at a corresponding position in myosin XI members, as shown in Table 3.

**Table 3:** **Sequence alignment of acidic residues in the motor domain and basic residues in GTD**.

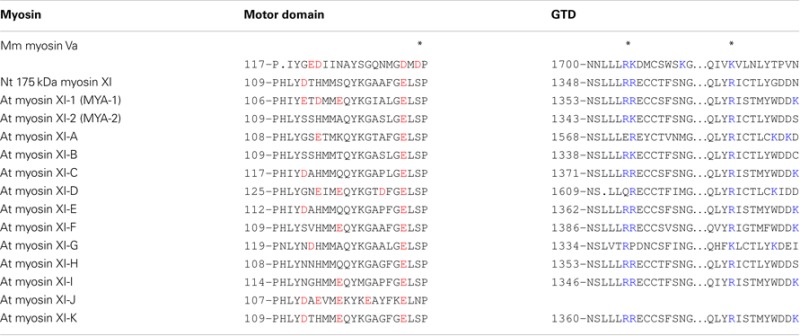

*Amino acid residues 117–137 and 1700–1788 of myosin Va are aligned with a representative selection of homologous sequences from plant myosin XI members. Acidic residues in the motor domain and basic residues in GTD are highlighted in red and blue, respectively. Asterisks show the critical amino acids for head–tail interaction of myosin Va. Mm, *Mus musculus*; Nt, *Nicotiana tabacum*; and At, *Arabidopsis thaliana**.

For full-length Nt 175-kDa myosin XI purified from cultured tobacco BY-2 cells, neither inactivation of ATPase activity in the presence of EGTA nor a folded conformation under electron microscopy was detected, indicating that the inhibited state is not equipped as a regulatory mechanism. The composition of acidic residues varies among At myosin XI members, suggesting the possibility of some myosin XI members forming an inhibited state conformation (Table 3).

The biological significance of the inhibited state is believed to be an efficient recycling of myosin molecules. Specifically, myosin V will release its cargo after it reaches the terminus of the actin track at the cell periphery, and take on the folded conformation of the inhibited state in order to be recruited to the initial point without much consumption of ATP.

In the case of plant cells, the significance of such a recycling system for myosin is uncertain, because cytoplasmic streaming is rotational and continuous. In plant cells, myosin XI recycling might be important for tip-growing cells or in local areas such as junctions with microtubules or the ER network, where myosin XI could release cargo from the actin track to the next step in the trafficking pathway.

## Concluding Remarks and Future Issues

As described in this review, plant myosin XI members are expected to be highly functional molecules equipped with various motile properties and regulatory systems.

In addition to their molecular properties, many unsolved questions remain unanswered. Most of the adaptor proteins that specifically link the myosin XI tail to cargo are unknown. Neither spatial nor temporal expression of each myosin XI member is completely understood at either the cellular or tissue level. The mechanism of how myosin XI members collaborate redundantly or distinctly at the correct time and place is also unclear. The existence and function of splicing variants are not yet understood (Peremyslov et al., 2011). Interactions with microtubule-based motor kinesins, a common regulatory mechanism for trafficking in animal cells, remain unidentified.

It is impossible to identify the biological function of myosin XI solely from biochemical and mechanical analyses. Similar cell biological analysis is inadequate for understanding the relationship between molecular properties and the biological function of myosin XI. To completely answer these questions, a combination of experimental approaches from multiple disciplines is required.

Because the last paragraph of this section is described based on the unpublished data, it may belong to the future issues. We have already cloned full-length cDNA of all plant myosin members from Arabidopsis, including myosin VIII. A wide range of analyses are in progress using these full-length DNAs. Biochemical and biophysical approaches have partially revealed various motile and enzymatic properties of several myosin members. Expression of full-length myosins fused with GFP has shown distinct intracellular localization and movement of individual myosin members in plant cells. A promoter assay has revealed distinct tissue-specific expression among myosin members. Although there is a long way to go, integration of the information from various approaches is enabling elucidation of the plant-specific actin–myosin system from the molecular level to a higher level.

## Conflict of Interest Statement

The authors declare that the research was conducted in the absence of any commercial or financial relationships that could be construed as a potential conflict of interest.
